# Thymol nanoemulsion promoted broiler chicken’s growth, gastrointestinal barrier and bacterial community and conferred protection against *Salmonella* Typhimurium

**DOI:** 10.1038/s41598-021-86990-w

**Published:** 2021-04-08

**Authors:** Doaa Ibrahim, Ahmed Abdelfattah-Hassan, M. Badawi, Tamer Ahmed Ismail, Mahmoud M. Bendary, Adel M. Abdelaziz, Rasha A. Mosbah, Dalia Ibrahim Mohamed, Ahmed H. Arisha, Marwa I. Abd El-Hamid

**Affiliations:** 1 Department of Nutrition and Clinical Nutrition, Faculty of Veterinary Medicine, Zagazig University, Zagazig, Egypt; 2 Department of Anatomy and Embryology, Faculty of Veterinary Medicine, Zagazig University, Zagazig, Egypt; 3 Biomedical Sciences Program, Zewail City of Science and Technology, University of Science and Technology, October Gardens, 6th of October, Giza, 12578 Egypt; 4 Department of Clinical Laboratory Sciences, Turabah University College, Taif University, P.O. Box 11099, Taif, 21944 Saudi Arabia; 5 Department of Microbiology and Immunology, Faculty of Pharmacy, Port Said University, Port Said Governorate, Egypt; 6 Faculty of Veterinary Medicine, Veterinary Educational Hospital, Zagazig University, Zagazig, Egypt; 7 Zagazig University Hospital, Zagazig, Egypt; 8 Department of Biochemistry, Zagazig Branch, Agriculture Research Center, Animal Health Research Institute, Zagazig, Egypt; 9 Department of Animal Physiology and Biochemistry, Faculty of Veterinary Medicine, Badr University in Cairo (BUC), Badr City, Cairo, Egypt; 10 Department of Physiology, Faculty of Veterinary Medicine, Zagazig University, Zagazig, Egypt; 11 Department of Microbiology, Faculty of Veterinary Medicine, Zagazig University, Zagazig, Egypt

**Keywords:** Microbiology, Physiology

## Abstract

The present study involved in vivo evaluation of the growth promoting effects of thymol and thymol nanoemulsion and their protection against *Salmonella* Typhimurium infection in broilers. One-day old 2400 chicks were randomly divided into eight groups; negative and positive control groups fed basal diet without additives and thymol and thymol nanoemulsion groups (0.25, 0.5 and 1% each). At d 23, all chicks except negative control were challenged with *S*. Typhimurium. Over the total growing period, birds fed 1% thymol nanoemulsion showed better growth performance even after *S*. Typhimurium challenge, which came parallel with upregulation of digestive enzyme genes (*AMY2A*, *PNLIP* and *CCK*). Additionally, higher levels of thymol nanoemulsion upregulated the expression of *MUC*-2, *FABP*2, *IL-10*, *IgA* and tight junction proteins genes and downregulated *IL-2* and *IL-6* genes expression. Moreover, 1% thymol nanoemulsion, and to lesser extent 0.5% thymol nanoemulsion and 1% thymol, corrected the histological alterations of cecum and liver postinfection. Finally, supplementation of 1% thymol, 0.5 and 1% thymol nanoemulsion led to increased *Lactobacilli* counts and decreased *S*. Typhimurium populations and downregulated *invA* gene expression postinfection. This first report of supplying thymol nanoemulsion in broiler diets proved that 1% nano-thymol is a potential growth promoting and antibacterial agent.

## Introduction

The quest for alternative natural products has intensified lately with more and more strict regulations regarding the antibiotic use as growth promoters in addition to the consumers need for antibiotics free poultry products^1^. Recently, the essential oils (EOs) have been gaining considerable interest due to their ability to improve the growth performance, gut health and intestinal integrity, strengthen the mucosal barrier and thereby limit the diseases challenges in poultry^2–4^. Besides, the antibacterial property of EOs has been well recognized^5,6^. Thymol, the main phenolic ingredient of thyme (*Thymus vulgaris*) essential oil, is among the reported plant compounds those are used in poultry nutrition as feed additives. It is used to enhance the performance parameters and feed utilization efficiency of poultry with emphasis on the digestion and metabolism as well as its potency to change the gut microbiota^7^. Recently, thymol was shown to play an important role in enhancing the intestinal barrier function and reducing the cytokine genes expression during inflammation^8^. Moreover, it possesses a strong antimicrobial efficacy against various pathogenic bacteria^9^.

Among the most common diseases occurring in poultry^10^ are those caused by the genus *Salmonella*. Specifically, *Salmonella enterica* serovar Typhimurium is one of the most common enteric pathogenic bacteria, which is known to cause serious economic losses to the poultry sector as well as being associated with foodborne outbreaks in humans due to the consumption of contaminated poultry products^11^. Most birds infected by *S*. Typhimurium don’t show clinical signs and remain asymptomatic for long periods. However, the occurrence of the clinical diseases in terms of reduced growth, loss of egg production and mortality have been observed in birds submitted to many stress conditions and young broiler chicks with an immature immune system^12^. *Salmonella* Typhimurium has been shown to survive and replicate within avian macrophages, which is essential for the full expression of its virulence^13^. Moreover, the invasin A (*invA*) is one of the most vital virulence genes that is used as a biomarker for *Salmonella* species, since it is found in the *Salmonella* outer membrane, *invA* is responsible for facilitating the entry into intestinal epithelial cells thus initiating the infection^13^. In addition, a distinct array of cytokines that are released in response to *Salmonella* infection contributes to the development of inflammatory reactions in the intestine. Also, *Salmonella* species possess effector proteins that modulate the structure and functions of the intestinal tight junctions (TJ)^14^.

From this point, a number of approaches for reducing the *Salmonella* colonization in poultry has been explored thus far, but with various degrees of success and with many drawbacks. These approaches include feeding chickens with competitive exclusion bacteria, bacteriophages, organic acids, oligosaccharides, antibiotics and vaccines^15,16^. In this direction, preparation of thymol nanoemulsion represents a promising antimicrobial alternative^17^, that can be a novel alternative approach, for reducing or preventing *Salmonella* infection in poultry for maximizing its performance without noticeable drawbacks. Owing to their nanometric size, the nanocarriers can promote the essential oils bioactivity, since they improve the cellular uptake and enhance the deep tissue penetration thus improving the site-specific controlled release of the active ingredients^18^.

To our best knowledge, there are no reports evaluating the growth promoting and protective effect of thymol nanoemulsion against *S*. Typhimurium infection in vivo. Therefore, the present study explored whether thymol nanoemulsion could have a positive impact than thymol, even after exposure to *S*. Typhimurium, on the growth performance, cecal microbiology, cecal and liver histological alterations and the transcription of genes encoding digestive enzymes, tight junction proteins (TJP) and cytokines in chickens. This study also aimed to develop an experimental model for *S*. Typhimurium challenge for evaluating the anti-*Salmonella* therapeutic potentials of thymol and thymol nanoemulsion with an effort to verify their efficiency in modulating the *Salmonella invA* virulence gene expression.

## Results

### Growth performance

The growth performance data throughout the experimental period (pre- and post-infection) is illustrated in Table 1. Dietary supplementation of thymol or thymol nanoemulsion at different levels did not influence the performance of broilers during d 0 to 10 (starter period).

**Table 1:** Effects of thymol and thymol nanoemulsion on growth performance of Ross broilers (d 1 to 42) and challenged with *Salmonella* Typhimurium at d 23 of age.

Groups	Starter (1 to 10 d old)			Grower (11 to 22 d old)			Finisher (23 to 42 d old)			Allover (1 to 42 d old)		
	FI, (g/bird)	BWG, (g/bird)	FCR	FI (g/bird)	BWG (g/bird)	FCR	FI, (g/bird)	BWG, (g/bird)	FCR	FI, (g/bird)	BWG, (g/bird)	FCR
NC	351^ab^	288^ab^	1.22^ab^	1495^bc^	885^d^	1.69^a^	2381^a^	1323^a^	1.80^e^	4229^b^	2496^ab^	1.69^d^
PC	351^ab^	287^ab^	1.22^ab^	1503^b^	892^ cd^	1.68^a^	1995^d^	900^ g^	2.22^a^	3848^f^	2079^ g^	1.85^a^
**Thymol, %**												
0.25	351^ab^	280^b^	1.25^b^	1498^bc^	896^ cd^	1.67^a^	1989^d^	929^f^	2.14^b^	3837^f^	2106^f^	1.82^b^
0.5	352^ab^	288^ab^	1.22^ab^	1467^c^	898^ cd^	1.63^b^	1964^d^	1008^e^	1.95^c^	3783^ g^	2195^e^	1.72^d^
1	354^a^	286^ab^	1.24^ab^	1529^ab^	938^b^	1.63^b^	2092^c^	1092^c^	1.92^d^	3974^d^	2315^c^	1.72^d^
**Thymol nanoemulsion, %**												
0.25	351^ab^	291^a^	1.21^a^	1505^b^	912^c^	1.65^b^	2088^c^	1062^d^	1.96^c^	3944^e^	2266^d^	1.74^c^
0.5	352^ab^	283^ab^	1.24^ab^	1552^a^	960^a^	1.62^bc^	2130^b^	1155^b^	1.85^e^	4035^c^	2398^b^	1.68^d^
1	348^b^	283^ab^	1.23^ab^	1529^ab^	959^a^	1.60^c^	2380^a^	1338^a^	1.78^ef^	4256^a^	2580^a^	1.65^e^
*P*-value	0.05	0.01	0.02	< 0.001	< 0.001	< 0.001	< 0.001	< 0.001	< 0.001	< 0.001	< 0.001	< 0.001
SEM	3.11	1.20	< 0.001	23.14	60.86	< 0.001	22.77	66.78	< 0.001	27.80	6.89	< 0.001

NC (negative control): birds fed basal diet; PC (positive control): birds fed basal diet and challenged with *S*. Typhimurium at d 23 of age; thymol 0.25, 0.5 and 1%: birds fed basal diet supplemented with 0.25, 0.5 and 1% thymol; thymol nanoemulsion 0.25, 0.5 and 1%: birds fed basal diet supplemented with 0.25, 0.5 and 1% thymol nanoemulsion. All groups except NC group were challenged with *S.* Typhimurium at d 23 of age. FI: feed intake; BWG: body weight gain; FCR: feed conversion ratio. ^a-g^Mean values with different letters in the same column differ significantly at *p* < 0.05.

The body weight gain (BWG) and feed conversion ratio (FCR) in grower and finisher periods were greatly affected by dietary thymol and thymol nanoemulsion addition in a dose dependent manner. During the grower period, broilers fed 0.5 and 1% thymol nanoemulsion had the most significant increase (*p* < 0.05) in BWG (about 7%) when compared to negative control (NC) group. Following experimental infection during the finisher period, the BWG was not impaired by *S.* Typhimurium challenge in thymol nanoemulsion groups, followed by groups fed thymol comparing with positive control (PC) group. Broilers fed 1% thymol nanoemulsion and NC group showed the most significant (*p* < 0.05) improvement in BWG and FCR (1338 and 1323 g and 1.78 and 1.80, respectively), followed by groups fed 0.5% thymol nanoemulsion (1155 g and 1.85), then 1% thymol (1092 g and 1.92) when compared with the PC group. Concerning the total growing period, the most prominent increase in BWG and decrease in FCR were observed in NC, 0.5% and 1% thymol nanoemulsion groups with no significant differences between NC and 1% thymol nanoemulsion groups.

### Gene expression analysis of digestive enzymes, tight junction proteins and barrier function

**Figure 1:**
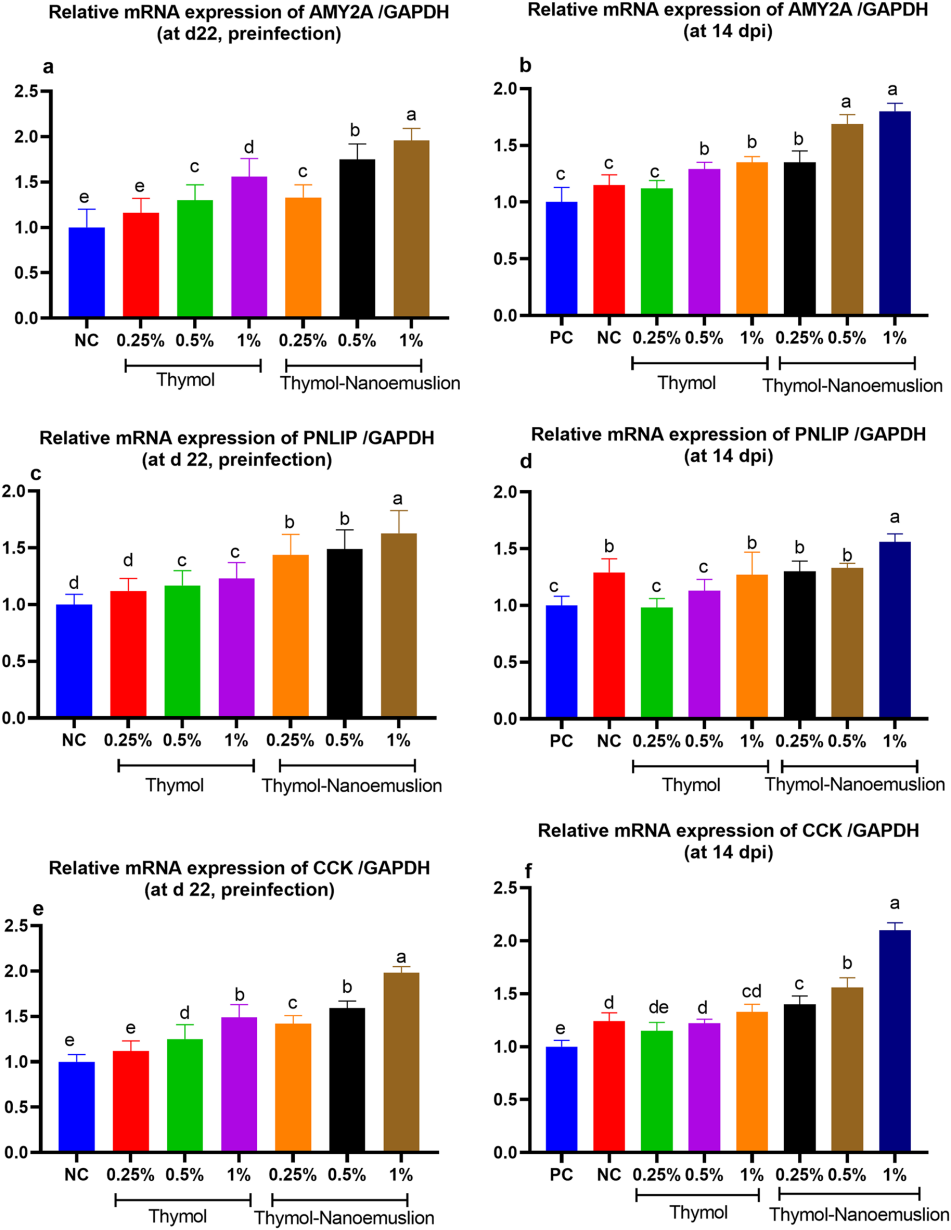
Levels of *AMY2A* (**a**,**b**), *PNLIP* (**c**,**d**) and *CCK* (**e**,**f**) mRNA expression in the pancreas of broiler chickens fed different levels of thymol and thymol nanoemulsion preinfection (d 22) and 14 days postinfection (14 dpi) with *S*. Typhimurium as were measured by RT-qPCR assay. Values are means with their SE in bars. At d 22 preinfection: NC (negative control) = birds fed basal diet, thymol 0.25, 0.5 and 1% = birds fed basal diet supplemented with 0.25, 0.5 and 1% thymol, thymol nanoemulsion 0.25, 0.5 and 1% = birds fed basal diet supplemented with 0.25, 0.5 and 1% thymol nanoemulsion. At 14 dpi: NC (negative control): birds fed basal diet; PC (positive control): birds fed basal diet and challenged with *S*. Typhimurium at d 23 of age; thymol 0.25, 0.5 and 1%: birds fed basal diet supplemented with 0.25, 0.5 and 1% thymol; thymol nanoemulsion 0.25, 0.5 and 1%: birds fed basal diet supplemented with 0.25, 0.5 and 1% thymol nanoemulsion. All groups except NC group were challenged with *S*. Typhimurium at d 23 of age. ^a–e^Means within the same column carrying different superscripts are significantly different at *p* < 0.05.

The mRNA expression of pancreatic alpha 2A amylase (*AMY2A*), pancreatic lipase (*PNLIP*) and cholecystokinin (*CCK*) genes was up regulated in response to dietary inclusion of higher levels of thymol or thymol nanoemulsion when compared with NC group preinfection and PC group postinfection. Notably, the most prominent effects were reported for thymol nanoemulsion groups (Fig. 1). Pancreatic *AMY2A* expression was up upregulated (increased by 0.75 and 0.96-fold preinfection (Fig. 1**a**) and increased by 0.69 and 0.80-fold postinfection (Fig. 1b) in 0.5 and 1% thymol nanoemulsion supplemented groups, respectively) when compared with control groups. Pancreatic *PNLIP* was mostly upregulated with 0.63-times preinfection (Fig. 1**c**) and 0.53-times posinfection (Fig. 1**d**) in 1% thymol nanoemulsion supplemented group when compared with control groups. The highest upregulation of *CCK* gene was observed in 0.5 and 1% thymol nanoemulsion groups (increased by 0.59 and 0.98-fold preinfection (Fig. 1**e**) and increased by 0.56 and two-fold postinfection (Fig. 1**f**), respectively comparing with the control groups).

**Figure 2:**
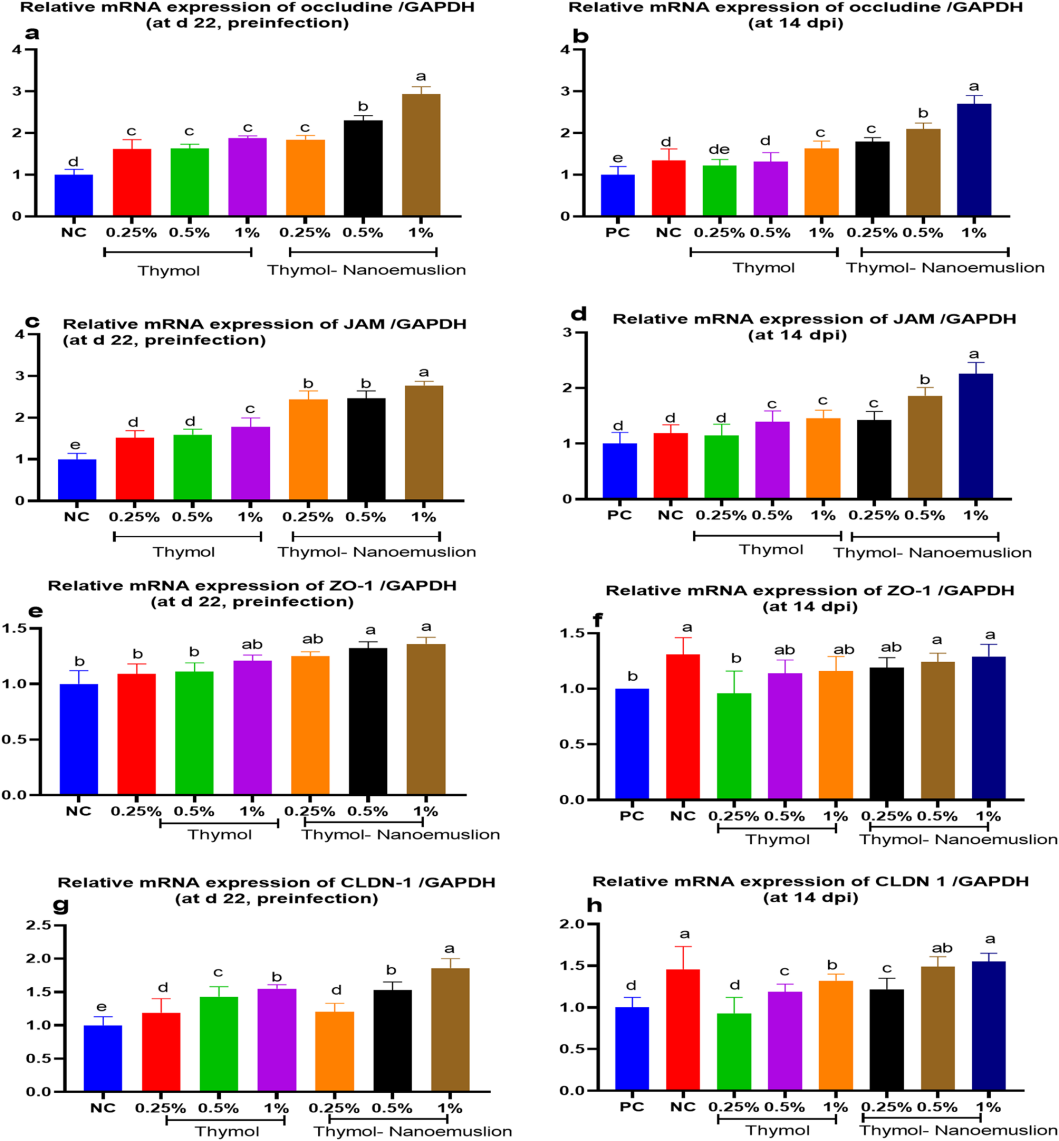
Real-time PCR analysis for occludin (**a**,**b**) junction adhesion molecule (*JAM*; **c**,**d**) zonula occludens (*ZO-*1; **e**,**f**) and claudins (*CLDN-1*; **g**,**h**) mRNAs expression in the cecal samples obtained from broilers fed a diet supplemented with different levels of thymol and thymol nanoemulsion preinfection (d 22) and 14 days postinfection (14 dpi) with *S.* Typhimurium. Data are presented as means ± SE. At d 22 preinfection: NC (negative control) = birds fed basal diet, thymol 0.25, 0.5 and 1% = birds fed basal diet supplemented with 0.25, 0.5 and 1% thymol, thymol nanoemulsion 0.25, 0.5 and 1% = birds fed basal diet supplemented with 0.25, 0.5 and 1% thymol nanoemulsion. At 14 dpi: NC (negative control): birds fed basal diet; PC (positive control): birds fed basal diet and challenged with *S*. Typhimurium at d 23 of age; thymol 0.25, 0.5 and 1%: birds fed basal diet supplemented with 0.25, 0.5 and 1% thymol; thymol nanoemulsion 0.25, 0.5 and 1%: birds fed basal diet supplemented with 0.25, 0.5 and 1% thymol nanoemulsion. All groups except NC group were challenged with *S*. Typhimurium at d 23 of age. ^a–e^Means within the same column carrying different superscripts are significantly different at *p* < 0.05.

Dietary supplementation of thymol or thymol nanoemulsion with higher levels significantly (*p* < 0.05) upregulated mRNA expression of the TJP genes including occludin ((Fig. 2a,b), junctional adhesion molecules (*JAM*) ((Fig. 2c,d), zona occludens-1 (*ZO-1*) ((Fig. 2e,f) and claudins-1 (*CLDN 1*) (Fig. 2,g,h) compared to the control groups. The group supplemented with 1% thymol nanoemulsion showed the most significant (*p* < 0.05) upregulation of occludin with 2.93 and 2.70-fold pre- and post-infection (Fig. 2a,b) and *JAM* with 2.77 and 2.26-fold pre- and post-infection **c**,**d**, respectively when compared with control groups.

**Figure 3:**
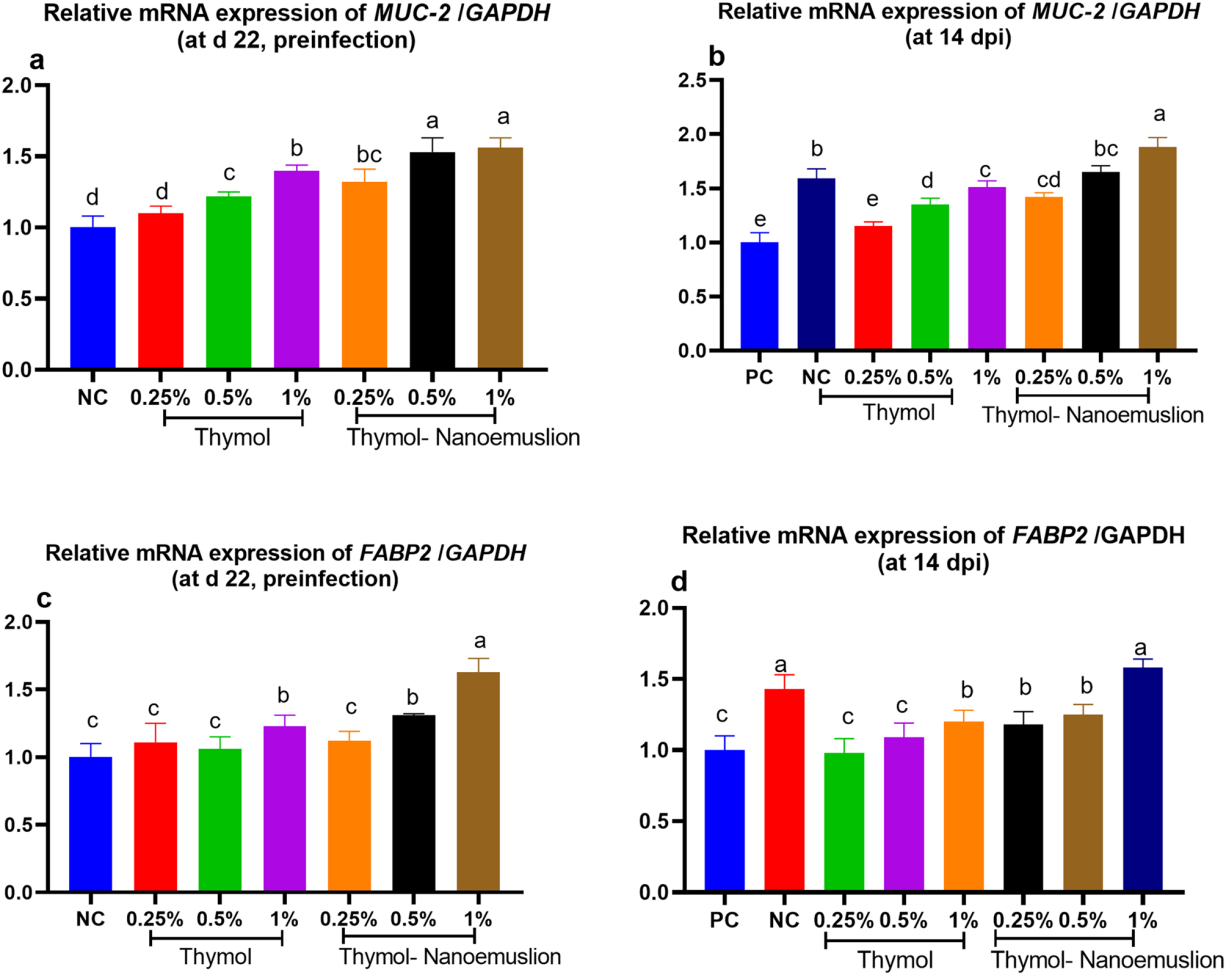
Relative mRNA expression levels of mucin-2 (*MUC*-2; **a**,**b**) and fatty acid binding proteins (*FABP2*; (**c**,**d**) in the spleen of broilers fed different levels of thymol and thymol nanoemulsion preinfection (d 22) and 14 days postinfection (14 dpi) with *S.* Typhimurium. Error bars represent SEM. At d 22 preinfection: NC (negative control) = birds fed basal diet, thymol 0.25, 0.5 and 1% = birds fed basal diet supplemented with 0.25, 0.5 and 1% thymol, thymol nanoemulsion 0.25, 0.5 and 1% = birds fed basal diet supplemented with 0.25, 0.5 and 1% thymol nanoemulsion. At 14 dpi: NC (negative control): birds fed basal diet; PC (positive control): birds fed basal diet and challenged with *S*. Typhimurium at d 23 of age; thymol 0.25, 0.5 and 1%: birds fed basal diet supplemented with 0.25, 0.5 and 1% thymol; thymol nanoemulsion 0.25, 0.5 and 1%: birds fed basal diet supplemented with 0.25, 0.5 and 1% thymol nanoemulsion. All groups except NC group were challenged with *S*. Typhimurium at d 23 of age. ^a–e^Means within the same column carrying different superscripts are significantly different at *p* < 0.05.

The upregulation of *MUC-2* (Fig. 3a,b) and *FABP2* (Fig. 3c,d) expression was more prominent (*p* < 0.05) in groups supplemented with thymol nanoemulsion than thymol. Postinfection, higher expression level of *MUC-2* was detected in groups supplemented with 0.5 and 1% thymol nanoemulsion, while the maximum upregulation of *FABP2* in this study was detected in 1% thymol nanoemulsion supplemented group in comparison to PC group.

### Modulation of cytokine gene expression

**Figure 4:**
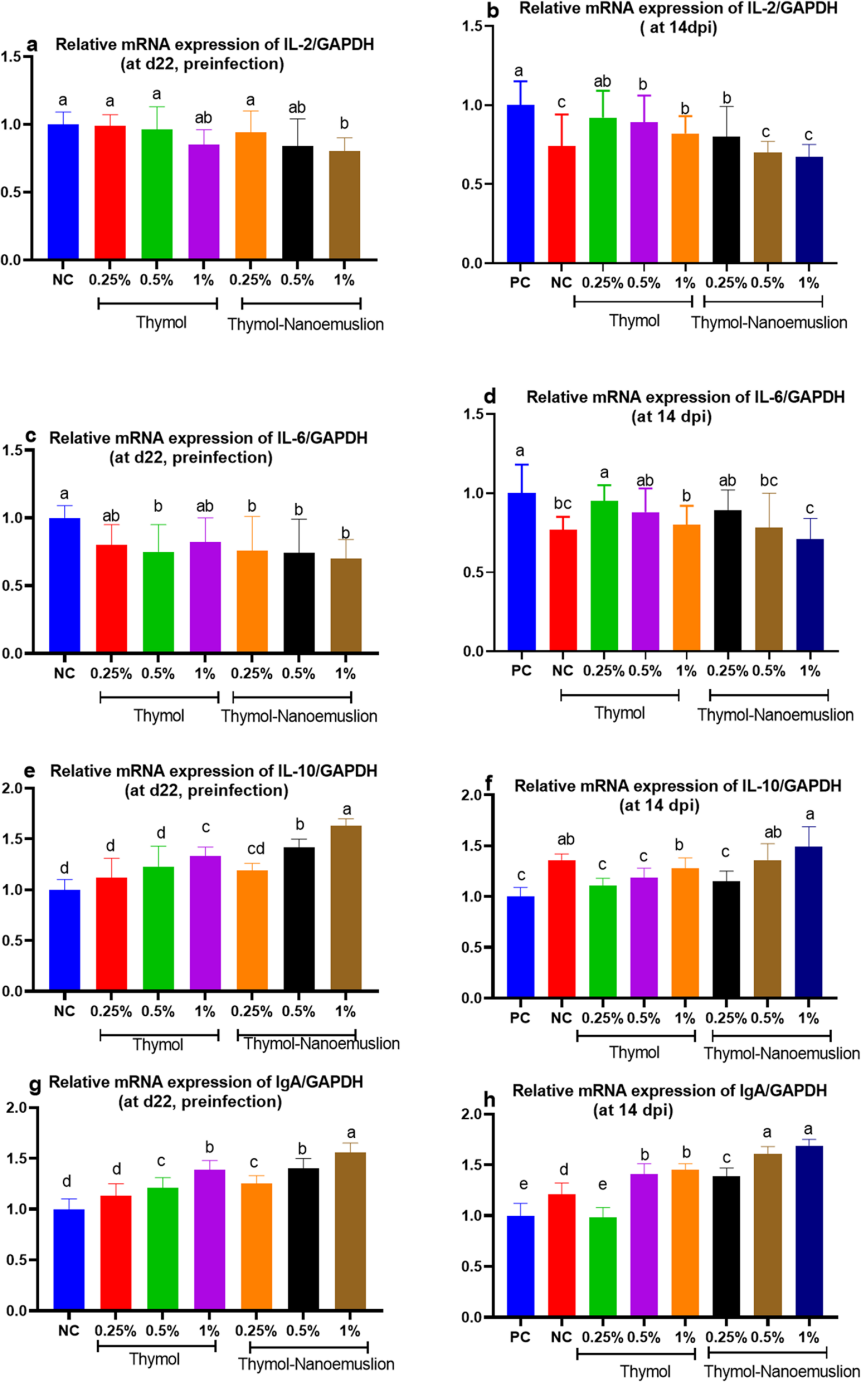
Relative mRNA expression levels of interleukin-2 (*IL-2*; **a**,**b**) interleukin-6 (*IL-6*; **c**,**d**) interleukin-10 (*IL-10*; **e**,**f**) and immunoglobulin A (*IgA*; **g**,**h**) in the spleen of broilers fed different levels of thymol and thymol nanoemulsion preinfection (d 22) and 14 days postinfection (14 dpi) with *S.* Typhimurium. Error bars represent SEM. At d 22 preinfection: NC (negative control) = birds fed basal diet, thymol 0.25, 0.5 and 1% = birds fed basal diet supplemented with 0.25, 0.5 and 1% thymol, thymol nanoemulsion 0.25, 0.5 and 1% = birds fed basal diet supplemented with 0.25, 0.5 and 1% thymol nanoemulsion. At 14 dpi: NC (negative control): birds fed basal diet; PC (positive control): birds fed basal diet and challenged with *S*. Typhimurium at d 23 of age; thymol 0.25, 0.5 and 1%: birds fed basal diet supplemented with 0.25, 0.5 and 1% thymol; thymol nanoemulsion 0.25, 0.5 and 1%: birds fed basal diet supplemented with 0.25, 0.5 and 1% thymol nanoemulsion. All groups except NC group were challenged with *S*. Typhimurium at d 23 of age. ^a–e^Means within the same column carrying different superscripts are significantly different at *p* < 0.05.

Preinfection, the transcriptional levels of interleukin-2 (*IL-2*) gene were not significantly different among various dietary treatments except for 1% thymol nanoemulsion when compared with the control group (Fig. 4a). Meanwhile, the most downregulation of *IL-2* gene was observed in 0.5 and 1% thymol nanoemulsion groups (about 0.5-fold reduction) compared to the PC group postinfection (Fig. 4b). Preinfection, the most pronounced downregulation of interleukin-6 (*IL-6*) was noticed in thymol nanoemulsion groups (Fig. 4c). Moreover, dietary supplementation of thymol or thymol nanoemulsion at different levels decreased the transcriptional levels of *IL-6* gene in a dose dependent manner compared to the PC group postinfection (Fig. 4d). The lowest transcriptional level was observed in 1% thymol nanoemulsion supplemented group with about 0.4-fold when compared with PC group.

Increased *IL-10* mRNA expression was observed in groups supplemented with different levels of thymol nanoemulsion and 1% thymol in comparison to the control groups (Fig. 4**e**,**f**).

Dietary supplementation of higher levels of thymol and thymol nanoemulsion significantly upregulated (*p* < 0.05) the expression levels of immunoglobulin A (*IgA*) when compared with the control groups even after infection (Fig. 4**g**,**h**).

### Cecal bacterial community composition

**Figure 5:**
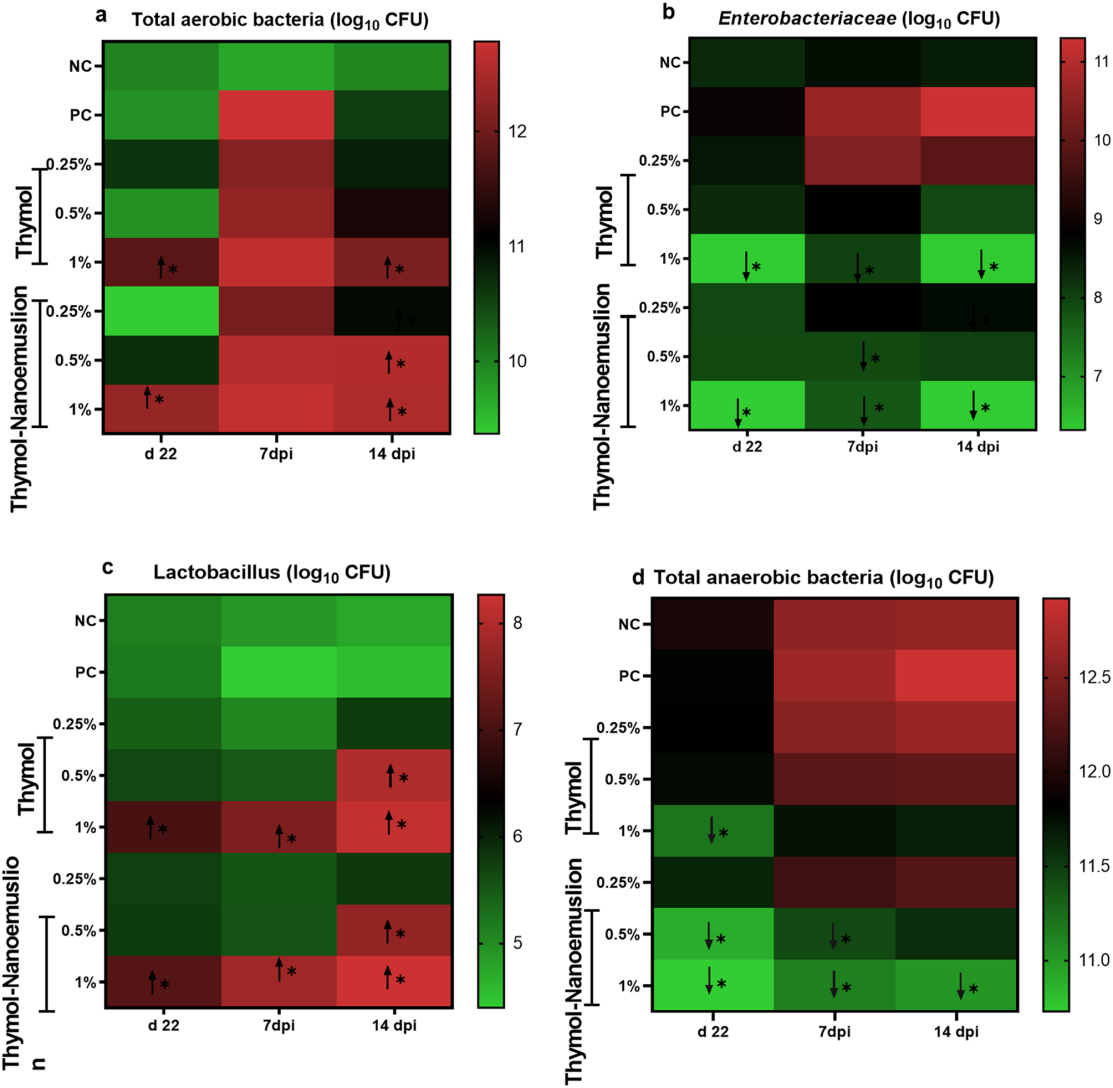
Heat map demonstrating the total aerobic bacterial (**a**), *Enterobacteriaceae* (**b**) and lactobacillus (**c**) total anaerobic bacterial (**d**) populations (log_10_ CFU) in the cecum of broiler chickens preinfection, d 22 and 7 and 14 days postinfection (7 and 14 dpi) with *S.* Typhimurium in response to supplementation with different levels of thymol and thymol nanoemulsion. *Red and green squares corresponded to significant counts increased (↑) and decreased (↓) relative to the control groups (*p* < 0.05). Other squares with no (*) denoted no significant changes in the bacterial counts among the experimental groups. At d 22 preinfection: NC (negative control) = birds fed basal diet, thymol 0.25, 0.5 and 1% = birds fed basal diet supplemented with 0.25, 0.5 and 1% thymol, thymol nanoemulsion 0.25, 0.5 and 1% = birds fed basal diet supplemented with 0.25, 0.5 and 1% thymol nanoemulsion. At 7 and 14 dpi: NC (negative control): birds fed basal diet; PC (positive control): birds fed basal diet and challenged with *S*. Typhimurium at d 23 of age; thymol 0.25, 0.5 and 1%: birds fed basal diet supplemented with 0.25, 0.5 and 1% thymol; thymol nanoemulsion 0.25, 0.5 and 1%: birds fed basal diet supplemented with 0.25, 0.5 and 1% thymol nanoemulsion. All groups except NC group were challenged with *S*. Typhimurium at d 23 of age.

As depicted in Fig. 5, dietary inclusion of 1% thymol and thymol nanoemulsion for 22 d significantly reduced (*p* < 0.05) *Enterobacteriaceae* population by 2–2.6 log_10_ CFU/g and anaerobic bacterial counts by 0.1–1.2 log_10_ CFU/g, also it increased the number of lactobacillus copies by 2 log_10_ CFU/g with respect to the control groups. Totally, 1% thymol and 1% thymol nanoemulsion groups had markedly higher total aerobic bacterial population and lower anaerobic bacterial count relative to the control groups (Fig. 5**a**,**d**).

At 7 days postinfection (dpi), *Enterobacteriaceae* and anaerobic bacterial counts were markedly decreased (*p* < 0.05) in 1% thymol and 0.5% and 1% thymol nanoemulsion groups in relation to the PC group (Fig. 5b,d). In principle, statistically significant (*p* < 0.05) rises in the lactobacillus counts were recorded for 1% thymol and 1% thymol nanoemulsion groups when compared to PC group (Fig. 5**c**). The pretreatment of challenged chickens with thymol and thymol nanoemulsion reduced cecal total aerobic bacterial count compared to the PC group (Fig. 5**a**). These values, although numerically lower, did not reach significant differences.

At 14 dpi, the *Enterobacteriaceae* and anaerobic bacterial counts were reduced in all treated groups in a dose dependent manner than the PC group with a trend towards significant differences for 1% thymol and 1% thymol nanoemulsion groups (Fig. 5**b,d**)*.* Compared with the PC group, the birds fed thymol and thymol nanoemulsion had considerable higher lactobacillus number of copies. However, the results were statistically significant for all supplemented groups except 0.25% thymol and 0.25% thymol nanoemulsion (Fig. 5**c**). Only 1% thymol and 0.5% and 1% thymol nanoemulsion groups had a markedly high number of total aerobic bacterial count (*p* < 0.05) than the PC group (Fig. 5**a**).

### Quantification of cecal *Salmonella* Typhimurium populations

**Figure 6:**
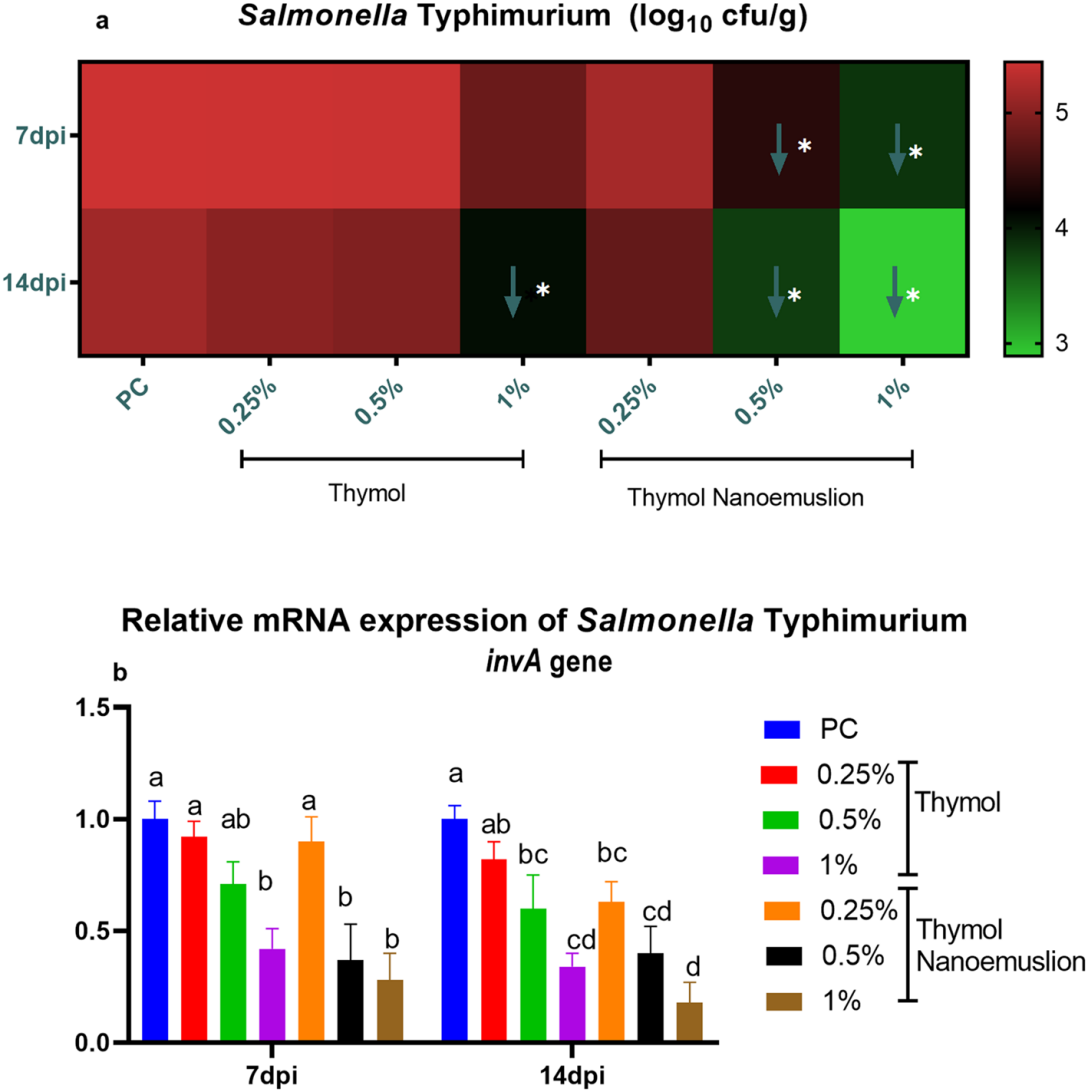
Quantification of cecal *S*. Typhimurium populations (**a**) and its *invA* mRNA (**b**) in response to thymol and thymol nanoemulsion supplementation at 7 and 14 dpi as were measured by real-time PCR assay. Data are expressed as means ± SE (error bars). Arrows corresponded to significant decrease (↓) relative to the PC group (*p* < 0.05). NC (negative control): birds fed basal diet; PC (positive control): birds fed basal diet and challenged with *S*. Typhimurium at d 23 of age; thymol 0.25, 0.5 and 1%: birds fed basal diet supplemented with 0.25, 0.5 and 1% thymol; thymol nanoemulsion 0.25, 0.5 and 1%: birds fed basal diet supplemented with 0.25, 0.5 and 1% thymol nanoemulsion. All groups except NC group were challenged with *S*. Typhimurium at d 23 of age. ^a–d^Means within the same column carrying different superscripts are significantly different at *p* < 0.05.

According to the results of quantitative analysis of *S.* Typhimurium in the cecal contents postinfection, lower *Salmonella* populations were detected in all treated groups with respect to the PC group and these counts were decreased steadily by time. At 7 dpi, the changes in the log_10_ values of *S.* Typhimurium CFU/g among majority of the supplemented groups showed no statistically significant differences when compared to the PC group. However, only significant lower (*p* < 0.05) log_10_ copies of *S.* Typhimurium populations were found in the cecal contents of both 0.5% and 1% thymol nanoemulsion-fed birds as was evidenced by decreased *Salmonella* counts by 1.03 and 1.6 log_10_ CFU/g, respectively (Fig. 6**a**). At 14 dpi, supplementation of 1% thymol and 0.5% and 1% thymol nanoemulsion significantly reduced (*p* < 0.05) *Salmonella* counts compared to the PC group. The most striking result was reported for 1% thymol nanoemulsion, where it greatly reduced *Salmonella* counts by 2.27 log_10_ CFU/g.

### Expression of *Salmonella* Typhimurium* invA* virulence gene

The relative mRNA expression levels of *S.* Typhimurium *invA* gene tended to be downregulated in birds provided with thymol and thymol nanoemulsion supplemented diet when compared to its levels in the PC birds postinfection, but the role of thymol nanoemulsion was more effective than thymol (Fig. 6**b**). Thymol and thymol nanoemulsion supplementation significantly reduced the *invA* gene expressions at 7 dpi (up to 0.42- and 0.28-fold) and at 14 dpi (up to 0.34- and 0.18-fold), respectively. The *invA* gene mRNA expression levels were lowered with increasing thymol and thymol nanoemulsion dosage. The most pronounced reduction (*p* < 0.05) in *invA* expression levels was observed in the cecal contents of 1% thymol and 0.5% and 1% thymol nanoemulsion supplemented birds at both time intervals, which reflect their protective mechanism against *S*. Typhimurium infection.

### Histological and histopathological outcomes

**Figure 7:**
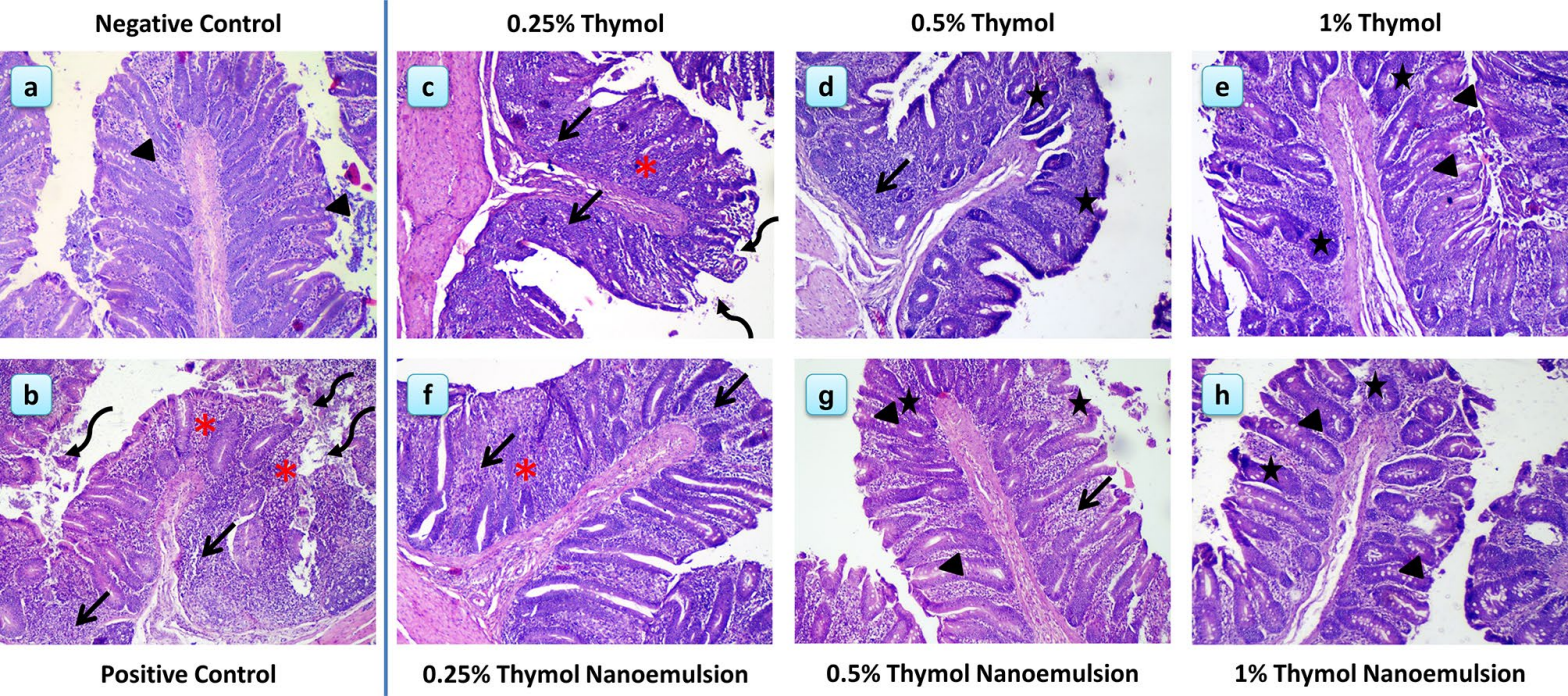
Histological and histopathological alteration of the cecum at 14 dpi. (**a**) negative control: birds fed basal diet without challenge with *S.* Typhimurium; (**b**) positive control: birds fed basal diet and challenged with *S*. Typhimurium ; (**c**) birds fed basal diet supplemented with 0.25% thymol, (**d**) birds fed basal diet supplemented with 0.5% thymol; (**e**) birds fed basal diet supplemented with 1% thymol; (**f**) birds fed basal diet supplemented with 0.25% thymol nanoemulsion; (**g**) birds fed basal diet supplemented with 0.5% thymol nanoemulsion; (**h**) birds fed basal diet supplemented with 1% thymol nanoemulsion. Inflammatory cells infiltration (arrow), degeneration (curved arrow), hemorrhagic foci and extravasated erythrocytes (red asterisk), hyperplastic enterocytes and active mucosal glands (star), and goblet cells (arrowhead). Stain H&E (magnification, 100 ×).

The cecum of negative control chicks showed normal structural integrity with preserved tissue architecture of the mucosa and submucosa (Fig. 7**a**). After 14 dpi, *S.* Typhimurium challenged chicks (positive control group) showed severe inflammation and degeneration of cecal villi with extensively desquamated epithelium. In addition, the villi became shorter and wider due to severe inflammatory cells infiltration and scattered hemorrhagic foci that completely distorted the crypt structure (Fig. 7**b**). Following supplementation with thymol, the cecal histopathological picture was improved with increasing thymol dose (Fig. 7**c–e**), in challenged chicks at 14 dpi. The number of damaged villi and the inflammatory cells infiltration became reduced with visible regeneration in villus epithelium, hyperplastic enterocytes and increased goblet cell activity, especially in the 0.5% (Fig. 7**d**) and 1% thymol (Fig. 7**e**) groups. Moreover, administering thymol nanoemulsion resulted in better improvement of cecal histological architecture and the effect was improved with increasing the dose of thymol nanoemulsion (Fig. 7**f–h**). In the 0.25% nanothymol group, there were less necrotic and sloughed epithelia compared to the 0.25% thymol group; however, it still showed thickened villi with inflammatory cells infiltration (Fig. 7f, compared to 7c, respectively). In the 0.5% thymol nanoemulsion group, there was a promotion in the cecal architecture with fewer leukocytic infiltration and enhanced epithelial hyperplastic response with increased number of goblet cells (Fig. 7**g**). Finally, the 1% thymol nanoemulsion group showed the least histopathological deterioration and the best histological architecture in all the studied groups following *S.* Typhimurium challenge; the cecal architecture showed a near normal appearance of villi height and thickness with slight leukocytic infiltration, epithelial and glandular hyperplastic changes with much higher goblet cells activity (Fig. 7**h**), compared to other challenged groups.

**Figure 8:**
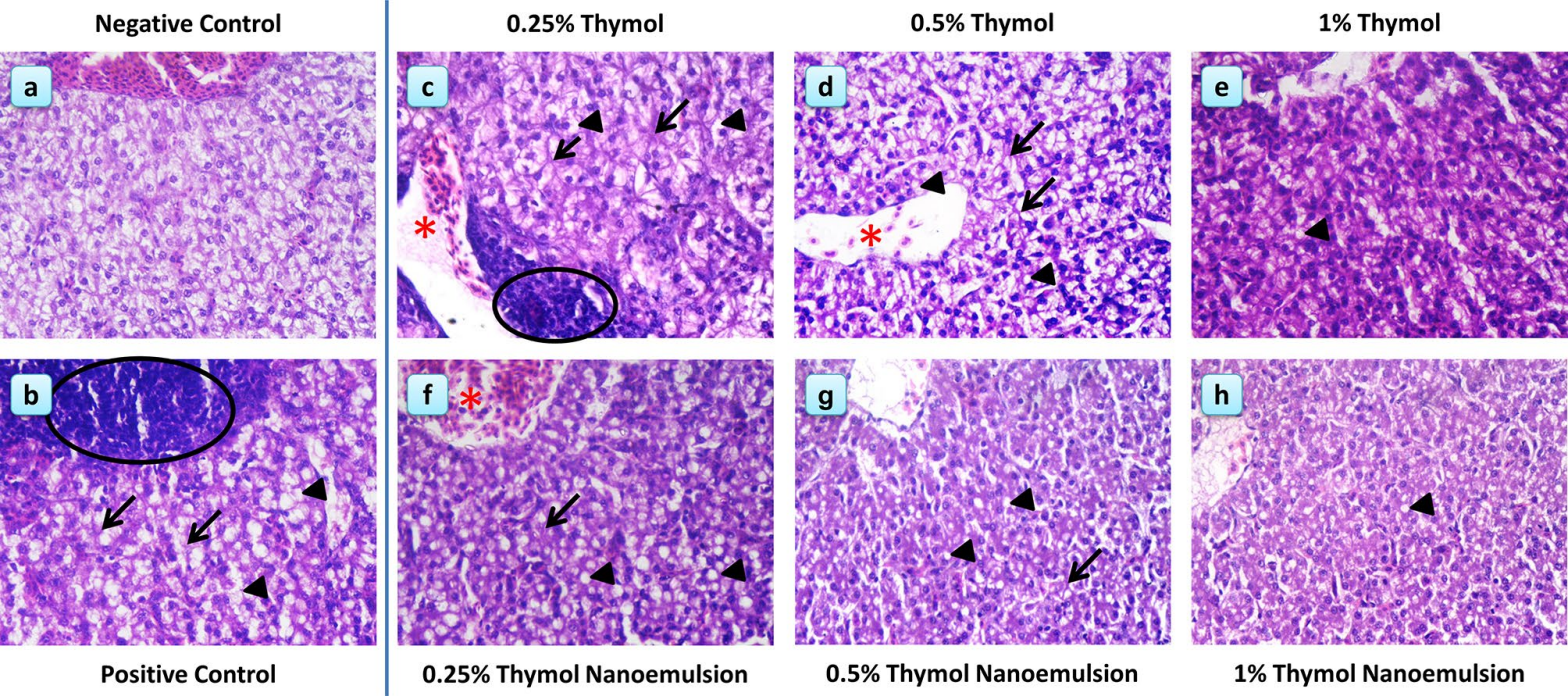
Histological and histopathological alteration of the liver at 14 days post-infection. (**a**) negative control: birds fed basal diet without challenge with *S.* Typhimurium; (**b**) positive control: birds fed basal diet and challenged with *S*. Typhimurium; (**c**) birds fed basal diet supplemented with 0.25% thymol, (**d**) birds fed basal diet supplemented with 0.5% thymol; (**e**) birds fed basal diet supplemented with 1% thymol; (**f**) birds fed basal diet supplemented with 0.25% thymol nanoemulsion; (**g**) birds fed basal diet supplemented with 0.5% thymol nanoemulsion; (**h**) birds fed basal diet supplemented with 1% thymol nanoemulsion. Focal leukocytic infiltration (circle), hydropic degeneration (arrow), vacuolation (arrow head), dilated hepatic blood vessels (red asterisk), and perivascular or interstitial leukocytic infiltration (arrows). Stain H&E (magnification, 400 ×).

The liver of the negative control group showed normal hepatic parenchyma and blood vessels (Fig. 8**a**). At 14 dpi, *S*. Typhimurium challenged chicks (positive control group) showed extensive lesions consisting of degenerative changes, vacuolations and large focal areas of leukocytic infiltration (Fig. 8**b**). Thymol supplementation resulted in improving the liver histological architecture at 14 dpi (Fig. 8**c–e**) and this was achieved with increasing thymol dosage. Hepatic vacuolations and perivascular and interstitial leukocytic infiltration were still visible in the 0.25% thymol group, but to a lesser extent compared to challenged non-supplemented chicks (Fig. 8**c**). Amelioration of these degenerative changes (vacuolation and leukocytic infiltration) was moderately achieved in the 0.5% thymol group (Fig. 8**d**); whereas in the 1% thymol group, the liver showed less vacuolations and leukocytic infiltration (Fig. 8**e**). However, using thymol nanoemulsion resulted in much better improvements in the histological architecture of the liver in challenged birds (Fig. 8**f–h**). In 0.25% thymol nanoemulsion supplemented group, there was a moderate degree of necrotic changes, hepatic vacuolation and leukocytic infiltration compared to the 0.25% thymol group (Fig. 8**f**, compared to 8**c**, respectively), and it was comparable to the 0.5% thymol group (Fig. 8**f**, compared to 8**d**, respectively). In 0.5% thymol nanoemulsion supplemented group, there was a clear promotion in the hepatic structure with less evident necrotic changes, hepatic vacuolation and leukocytic infiltration (Fig. 8**g**) and this was comparable to the 1% thymol group (Fig. 8**e**) and better than the 0.5% thymol group (Fig. 8**d**). Finally, the 1% thymol nanoemulsion group showed the least histopathological deterioration of the liver in all studied groups, following *S*. Typhimurium challenge, as it showed an apparent normal hepatic parenchyma with minute vacuolations in fewer number of hepatocytes (Fig. 8**h**).

## Discussion

*Salmonella* Typhimurium infection via foodborne transmission is considered a major public health issue in both developed and developing countries and it is still among the common causative agents of infectious poultry diseases. Therefore, reducing *Salmonella* populations in chickens can potentially reduce the contamination of poultry meat and its products and protect against food-borne salmonellosis. While screening the beneficial effects of essential oils and their purified components as feed additives in poultry, thymol is recognized to be a promising and safer alternative than growth promoting antibiotics with strong antimicrobial and anti-inflammatory properties^19^. The development of novel nano-delivery system for thymol as thymol nanoemulsion will increase the stability and absorption of thymol along the gastrointestinal tract in addition to its positive effects on the broiler's growth and health. Previous scientific research demonstrating these activities have been done in vitro^20^. Nevertheless, there are few in vivo studies reporting the effects of thymol in broiler chickens^21^ and, to our best knowledge, there are no reports available in the literature concerning the in vivo use of thymol nanoemulsion as a growth promoter and for protection of chickens against *S.* Typhimurium infection.

In the current study, although dietary thymol and thymol nanoemulsion supplementation had no evident effects on broilers gain or FCR during starter period, the positive effect of dietary thymol nanoemulsion inclusion on broiler’s performance during grower and finisher periods was clear. Allover the growing period, groups fed 0.5 and 1% thymol nanoemulsion had the highest BWG and better FCR even after *S.* Typhimurium challenge. In line with the increased growth rate and feed utilization in these groups, the expression of genes encoding digestive enzymes (*AMY2A*, *PNLIP* and *CCK*) were also upregulated. It was previously proven that thyme essential oils (1 g/kg) had positive effects on broiler growth, nutrient utilization and intestinal microflora^22,23^. The possible mechanism of essential oils on growth performance could be attributed to the increased digestibility of feed by stimulation of endogenous enzymes and regulation of the gut microbial flora^24^. Moreover, it has been reported that phytogenic compounds can regulate the gene expression profiles of ileal mucosa^25^ and stimulate digestive secretions for improving nutrient digestibility^26^. It was proven that dietary thyme oil increased the digestive enzymes and improved the nutrient utilization of broilers^27^. However, upregulating the digestive enzyme genes expression following thymol and thymol nanoemulsion was not studied till now. The boosting role of thymol nanoemulsion on broilers performance could be explained by enhancing the bioavailability and bioactivity of thymol, since thymol nanoemulsion allow a deeper tissue penetration and an easier cellular uptake in the gastrointestinal tract, which leads to efficient upregulation of the digestive enzymes’ genes. Furthermore, better broiler’s performance following dietary inclusion of thymol nanoemulsion even after exposure to *S.* Typhimurium infection could be attributed to lowered infection severity.

Intestinal mucosal barrier plays an important role in the absorption of nutrients, electrolytes and water. Besides, it protects the gut from invasion of enteric pathogens and prevents the leakage of proinflammatory molecules through the intestinal mucosal to the circulatory system^28^. The intestinal barrier is regulated by TJP that consist of several unique proteins including occludin, zona occludens-1, claudins-1 and JAM and those are essential for establishing intact physical barrier between the intestinal epithelial cells^29^. In the pathogenesis of many inflammatory diseases, disturbance in the production and formation of TJ complexes occurs^30^. The disruption of TJP could lead to reducing nutrient absorption, increasing permeability to luminal antigens, bacteria translocation, sustained inflammation and tissue damage^31^. In this regard, dietary supplementation of thyme essential oil has been previously reported to enhance the intestinal integrity and strengthen the mucosal barrier^32^. In the present study, higher concentrations of thymol nanoemulsion greatly upregulated genes encoding occludin, zona occludens-1, claudins -1, JAM, MUC-2 and FABP2 controlling the barrier functions even after experimental infection, and it was better than thymol. Consistent with our results, increased TJP gene expression and improved intestinal barrier function were observed in thymol and carvacrol-treated broilers challenged with *Clostridium. perfringens*^33^. Interestingly, mucin signifies the first line of immune defense in the gastrointestinal tract and enhancement of its secretion could be beneficial in preventing invasion of pathogens and toxins^34^. While lower expression of *MUC-2*, controlling mucin production, in the challenged untreated group can be related to potentiating gut inflammation. Additionally, inflammatory lesions can lessen goblet cells secreting mucin, prevent regeneration of mucosal layer and enhance further infection, bacterial translocation and intestine inflammation^35,36^. Fatty acid binding proteins (FABP) harmonize cells lipid responses and are recognized to be contributing to both inflammatory and metabolic pathways^37^. Herein, increasing expression levels of *FABP-2* post infection is needed for the recovery of dysbacteriosis and barrier failure^38^. Moreover, IgA is the most important immunoglobulin and its higher concentration at the mucosal sites plays an effective role in inhibiting infection^39^. Upregulation of *IgA* was notably observed in groups supplemented with higher levels of thymol and thymol nanoemulsion indicating good immune response of birds.

Cytokines contribute to the development of anti-inflammatory reactions and they are specialized for the priming of an adaptive response. In chickens, the T-helper type 1 cells produce the cytokine IL-2, which is crucial for various biological effects on many immune cells including the functional activation of the cells of the innate immune response^40^. IL-6 mediates proinflammatory responses and participates in the initial host's immune defense against pathogens in chicken^41,42^. The pathological process caused by *Salmonella* in chickens triggers the differential expression of certain genes encoding proinflammatory cytokines or interleukins thus activating the intestinal inflammatory responses^43^. The current results showed that dietary thymol or thymol nanoemulsion supplementation downregulated *IL-2* and *IL*-*6* genes expression levels with a remarkable effect for thymol nanoemulsion than thymol. The more reduction of proinflammatory cytokines in the thymol nanoemulsion groups even after *S.* Typhimurium infection indicated its strong anti-inflammatory efficacy. This could be due to the uniformly dispersed nano-droplets of thymol nanoemulsion, which easily penetrate and disrupt the microbial membrane^20^. Beside the important roles in immunity, cytokines were also demonstrated to affect TJ as pro-inflammatory cytokines could induce disruption of TJ^44^. In the present study, increasing levels of dietary thymol nanoemulsion resulted in suppressing the *IL-2* and *IL-6* genes expression, which was in line with our results of enhanced expression of intestinal barrier related genes.

The microbiota contributes to the development and maintenance of the intestinal epithelial barrier as well as the development of the immune system and competition with pathogenic microorganisms. In the present study, dietary thymol and thymol nanoemulsion altered the microbiological profile of total aerobic and anaerobic bacteria, lactobacillus and *Enterobacteriaceae* in the cecum of birds at d 22 (preinfection) and at d 30 and 42 (postinfection). In healthy birds, commensal bacterial communities colonized the gastrointestinal tract by adhesion and form a protective layer (biofilm) covering the surface of the mucosal epithelium. This layer is consisting of a variety of symbiotic microbial communities, which block the colonization of the intestine by pathogenic microorganisms^45^, the process of which is called “competitive exclusion”. During the invasion of the intestinal epithelium by infectious microbes possessing the most powerful pathogenicity factors, the intestinal epithelial barrier disintegrates and the number of opportunistic and pathogenic microflora sharply grows, but that of the symbiotic microorganisms declines^46^. With respect to our control groups, the cecal *Enterobacteriaceae* and anaerobic bacterial populations were reduced and the number of lactobacillus loads was increased in groups fed different concentrations of thymol and thymol nanoemulsion in a dose dependent manner even after infection with *S.* Typhimurium. Similarly, supplementation of thymol increased the ileum lactobacilli in broiler chickens after *Clostridium perfringes* infection^47^. Moreover, the higher abundance of lactobacilli counts in the thymol treated groups was in concordance with earlier in vivo studies^21,47^. A higher lactobacillus count was recently reported in fecal microbiota of chickens fed thymol microencapsulated blend compared to the control group^48^. The increase in the lactobacillus counts accompanied with the decrease in the anaerobic bacterial population may be attributed to the emergence of the competitive advantage in *Lactobacillaceae* over the obligate anaerobes in the caecum. Domination of lactobacillus indicated that dietary supplementation of thymol and thymol nanoemulsion, especially at higher doses yielded a positive effect on the growth and intestinal health of poultry. Indeed, *Thymus vulgaris* exhibited wide spectrum of antibacterial activities against MDR *Enterobacteriaceae* isolates^49^. A previous study has proved the in vitro antibacterial activities of thymol on different enteric bacteria including pathogenic ones (*Salmonella* species, *C. perfringens* and *E. coli*)^33^. Our present in vivo observations are in accordance with those previous in vitro studies proving the use of thymol as a sturdy antimicrobial agent. Notably, an earlier in vitro study has demonstrated that thymol nanoemulsion have more critical effects on inhibition of bacterial growth when compared to thymol^20^. In a recent study, *Enterobacteriaceae* counts showed a decreasing trend upon supplementation of chicken diet with microencapsulated blends of natural identical essential oils including thymol^48^, these results consistently support our findings. The increased antibacterial activities of thymol nanoemulsion could be comprehended by the fact that nanodroplets can easily penetrate and directly disrupt the bacterial membranes^50^.

The proliferation of intestinal pathogens frequently results in chronic inflammatory responses that reduce the poultry productivity with a high risk of poultry products contamination. *S*. Typhimurium infection in poultry has been demonstrated to increase mortality and gut lesions and it is responsible for gastroenteritis that is associated with foodborne disease in humans. The results of quantitative analysis of *S*. Typhimurium in the cecal contents post challenge indicated that supplementation with thymol and thymol nanoemulsion consistently decreased *Salmonella* populations with respect to PC group. Interestingly, dietary supplementation of thymol nanoemulsion significantly reduced *Salmonella* loads at 7 dpi with no pronounced effect for thymol during this period. Meanwhile, thymol nanoemulsion inclusion resulted in more reduction of *Salmonella* loads, especially at a higher concentration comparable to a moderate effect for 1% thymol at 14 dpi. This corroborates the data presented by other investigators^51^, where the highest concentration of thymol (4 g/kg) demonstrated some protection for chicks against *Salmonella* Enteritidis and prevented further mortalities. This may be attributed to degradation of the cell wall, damage to the cytoplasmic membrane and membrane proteins, leakage of cell contents, coagulation of cytoplasm, and depletion of the proton motive force^52^. Previous observations had shown that thymol nanoemulsion exhibited in vitro potential antibacterial activities^20^. Therefore, in this study, thymol nanoemulsion demonstrated a better efficacy to control *S*. Typhimurium challenge for the first time in broiler chickens providing a link between its activities in vitro and in vivo.

*Salmonella* pathogenicity is expressed in three ways; invasion, intracellular survival and colonization and it depends on many virulence factors. *Salmonella* uses virulence factors for entering the intestinal epithelium and surviving in the mucosal macrophages with a consequence of causing an acute inflammatory process^53^. Therefore, an antivirulence strategy is gaining great interest as an alternative method for controlling infection. Natural antivirulence agents such as plant compounds can influence virulence factors controlling pathogenesis^54–56^.

Herein, the protective effects of thymol and thymol nanoemulsion supplementation against the infection was supported by determining the expression of *invA* gene, which is known to play a critical role in *Salmonella* virulence in response to thymol and thymol nanoemulsion supplementation. Our reverse transcription quantitative PCR (RT-qPCR) results indicated that supplementation of 1% of thymol and thymol nanoemulsion significantly reduced the *invA* gene expressions at 7 dpi (up to 0.42- and 0.28-fold) and 14 dpi (up to 0.34- and 0.18-fold), respectively. In many in vitro studies, there was a documentation of the potential use of thymol to control *Salmonella* colonization by downregulating the transcription of its critical virulence genes. An earlier in vitro study described that thymol reduced cell invasion of *S*. Typhimurium by reducing its virulence gene expression^57^. In another recent study, thymol could downregulate genes involved in *S*. Typhimurium chemotaxis, motility, and virulence^58^. In an in vivo study^59^, thymol effectively protected mice against *S*. Typhimurium induced pathological damages via inhibition of the type III secretion system-dependent virulence properties. More importantly, the ability of thymol nanoemulsion to affect the *Salmonella* virulence gene expression in vivo has not yet been reported.

Enteric infection with *S.* Typhimurium is accompanied with colonization in the intestine (especially cecum) and liver causing extensive degenerative lesions in broiler chicks^60,61^. This was evident in our study since the challenged chicks showed degenerative changes and extensive leukocytic infiltration in both liver and cecum. In the latter, it caused disrupted mucosal barrier via inducing desquamated epithelium, loss of mucin-producing goblet cells and overall distorted villi shape, which became shorter and wider. Similar findings were previously reported^62,63^. This mucosal disruption and inflammation leads to interference with intestinal function, facilitates the progression of *S.* Typhimurium colonization and leads to reduction in bird’s body weight gain^64,65^.

Following supplementation of thymol nanoemulsion and thymol, there was a dose-dependent improvement in the histopathological architecture of the liver and cecum. The best results in this study were obtained in the 1% thymol nanoemulsion group, followed by the 0.5% thymol nanoemulsion and 1% thymol groups. The main features of cecal improvement comprised restored epithelial barrier and increased number of goblet cells with hyperactive mucosal glands, which support the gene expression findings of tight junction proteins and barrier function (upregulated *MUC-2* and *FABP2* and TJP genes). Similar improvements in broilers’ barrier functions and goblet cells number and function were achieved following the use of Zinc^64^, dietary clays^63^, encapsulated sodium butyrate^65^, *Pediococcus acidilactici*, mannan-oligosaccharide, butyric acid^66^ and L-arginine^67^ in *S.* Typhimurium challenged broiler chicks. An additional feature of cecal improvement is the reduced inflammatory cells infiltration in cecal mucosa, which is consistent with the modulation of cytokines gene expression (reduced *IL-2*, *IL-6* and increased *IL-10* genes expression). These histopathological findings correlate with the growth findings, where the 1% thymol nanoemulsion group was comparable to the non-challenged control group in terms of birds BWG and FCR and correlate with cecal *S.* Typhimurium population counts, where the lowest post-challenge *Salmonella* counts were obtained in the 1% thymol nanoemulsion group. Previous reports showed that thyme oil or thymol could improve growth performance and reduce cecal colonization following challenge with *Salmonella* spp.^51,68,69^ or *Clostridium perfringens*^47,70^. In addition, different phytogenic feed additives, including thymol, can be used as alternatives to antibiotics in broiler chicks challenged with *S.* Typhimurium^71^. Also, the liver showed less necrosis and near normal parenchyma with reduced perivascular and interstitial leukocytic infiltration in the 1% thymol nanoemulsion group, followed by the 0.5% thymol nanoemulsion and 1% thymol groups. Thymol was previously reported to protect the liver antioxidant contents and led to improved growth performance in broiler chicks^72^. In addition, an essential oil blend, containing thymol, reduced liver colonization by *Salmonella* spp*.* with no differences in the growth performance between oil treated and control broiler chicks^73^.

## Conclusion

The administration of 1% thymol nanoemulsion led to positive transcriptional modifications of broiler’s digestive enzymes. Moreover, the integrity of intestinal TJ barrier was positively maintained, and this was evidenced by upregulation of *MUC-2* and *IgA* genes and downregulation of *IL-2* and *IL-6* genes suggesting suppression of inflammatory reaction induced by *S*. Typhimurium. This was accompanied by corrected histopathological picture of both the cecum and liver. An additional evidence supporting the anti-*Salmonella* effect of thymol nanoemulsion was the decrease in *S*. Typhimurium loads and virulence at 14 dpi. Finally, our results recommend the use of 1% thymol nanoemulsion as a promising candidate with growth promoting and anti-virulence properties for the control of *S*. Typhimurium infection, which is an additional benefit for consumer's health.

## Methods

### Thymol extract and thymol nanoemulsion formulation

Thymol (EOC, Mol. Wt. 50,000), sodium alginate (medium viscosity, A-2033), polyoxyethylene (20) monooleate (Tween 80, food grade) were purchased from Sigma-Aldrich (St. Louis, MO, USA). For preparation of the oil phase, sodium alginate was dissolved in hot water at 70 °C with a continuous stirring until it was completely dissolved. A coarse or primary emulsion was made by mixing the sodium alginate solution as an aqueous phase and thymol essential oil (1% v/v) as a lipidic phase plus tween 80 (1% v/v) as a surfactant with a laboratory digital Ultra-Turrax mixer (IKA, Germany) at 3400 rpm for 2 min^74^. Ultra-pure water was used in all preparations. After that, the coarse emulsion was homogenized at 10,000 rpm for 15 min until nanoemulsion solution was formed. This mixture was sonicated using an Ultrasonicator (Bandelin SONOPULS 2200, Germany) for 10 min at 700 W.

### Characterization of thymol nanoemulsion

**Figure 9:**
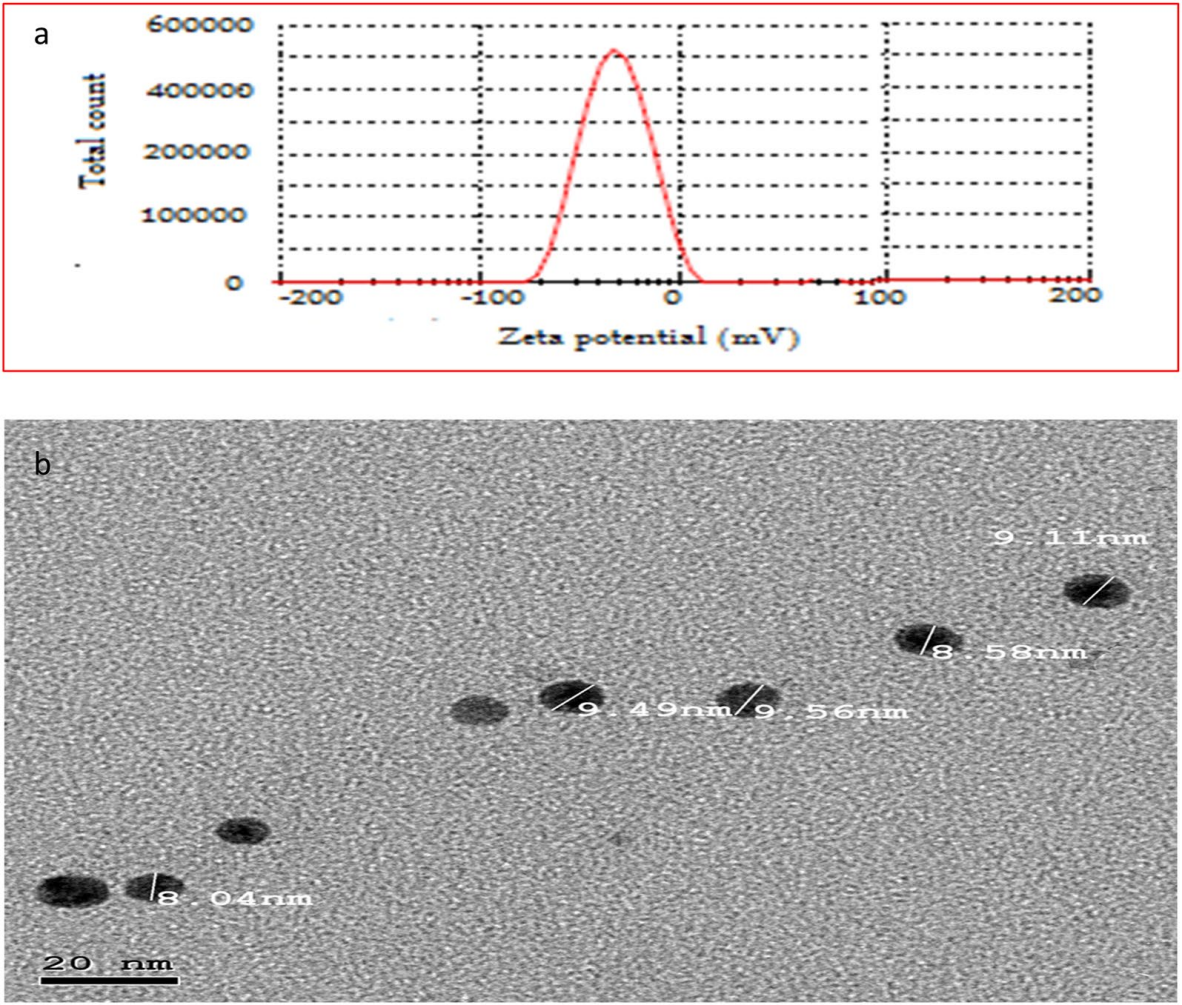
Zeta potential (**a**) and transmission electron microscopy (TEM, **b**) of thymol nanoemulsion.

The size, morphology, and stability of the synthesized thymol nanoemulsion were characterized using Zeta potential measurements, where thymol nanoemulsion presented a negative average zeta potential of − 25 mV (Fig. 9**a**) and transmission electron microscopy (TEM) (Fig. 9**b**) at National Center for Radiation Research and Technology (NCRRT), Atomic Energy Authority, Egypt.

### Birds, experimental design and diet

The experiment was carried out on a total of 2400 one-day-old male Ross 308 boiler chicks purchased from a local commercial hatchery farm. On arrival, the birds were initially checked to be free from any *Salmonella* spp. via bacteriological examination of cloacal swabs and fecal samples following the International Organization for Standardization (ISO) 6579 standards^75^. The birds were individually weighed and randomly assigned into eight dietary experimental treatment groups (ten replicates/group and 30 birds/replicate). Treatments were set as following: two control groups; NC birds received a control diet without thymol or thymol nanoemulsion and were not challenged and PC birds received a control diet without additives and were challenged at d 23 of age with *Salmonella* Typhimurium strain, birds in groups 2, 3 and 4 received a control diet supplemented with thymol at concentrations of 0.25, 0.5 and 1%, respectively and birds in groups 6, 7 and 8 were fed a control diet supplemented with thymol nanoemulsion at concentrations of 0.25, 0.5 and 1%, respectively. The supplemented diets were provided starting from the first day of life. Feed and drinking water were provided ad libitum throughout the experiment period of 42 days. All diets were offered in the mash form and the control diets (starter, grower and finisher) were formulated according to nutrition specification of Ross broiler handbook^76^ as shown in Table 2. The chemical analyses (moisture, crude protein, ether extract and crude fiber) of all feed ingredients were conducted using the standard method as recommended by Association of Official Analytical Chemists, AOAC^77^.

**Table 2:** The ingredients and nutrient content of basal diet.

Ingredient, %	Starter (1–10 days)	Grower (11–22 days)	Finisher (23–42 days)
Yellow corn	58	60.8	64.8
Soybean meal, 48%	35	31.3	26.3
Soybean oil	2.2	3.2	4.2
Calcium carbonate	1.2	1.2	1.2
Calcium diphasic phosphate	1.5	1.5	1.5
Common salt	0.3	0.3	0.3
Premix^a^	0.9	0.9	0.9
L-Lysine HCL, 78%	0.35	0.3	0.3
DL-Methionine, 99%	0.25	0.2	0.2
Choline chloride	0.20	0.20	0.20
Anti-mycotoxin	0.10	0.10	0.10
**Calculated composition**			
Metabolizable energy (Kcal/Kg)	3106	3103	3203
Crude protein, %	23.01	21.5	19.50
Ether extract, %	4.63	5.6	6.74
Crude fiber, %	2.63	2.56	2.46
Calcium, %	1.19	1.19	1.18
Available phosphorous, %	0.53	0.51	0.48
Lysine, %	1.45	1.30	1.17
Methionine, %	0.58	0.52	0.49

^a^Vitamin premix supplied per kilogram of diet: retinol, 10,000 IU; tocopheryl acetate, 70 mg, cholecalciferol, 6000 IU; menadione, 2.5 mg; riboflavin, 7 mg; thiamine, 4 mg; pantothenate, 12 mg; niacin, 50 mg; folate, 3 mg; pyridoxine, 6 mg; biotin, 300 μg; cyanocobalamine, 15 μg; Fe (sulphate), 30 mg; Cu (sulphate), 14 mg; Se (selenate), 0.3 mg; I (iodide), 1.20 mg; Zn (sulphate and oxide), 120 mg; Mn (sulphate and oxide), 100 mg.

### Experimental infection by Salmonella Typhimurium

#### Challenge inoculum

*Salmonella* Typhimurium strain used in this experiment was previously isolated from the visceral organs of freshly dead broiler chickens according to a previous study of one of the co-authors^78^. The strain was retrieved from the suspensions stored at − 80 °C and cultured onto brain heart infusion (BHI) broth (Oxoid, UK) for 24 h at 37 °C. Subsequently, the prepared culture was streaked onto xylose lysine deoxycholate agar (Oxoid, UK) and incubated for 24 h at 37 °C. One colony was transferred to sterile BHI broth and incubated at 37 °C for 2 h. The challenge inoculum was prepared by diluting the suspension appropriately in BHI broth to give a final viable cell concentration of 3 × 10^6^ CFU/mL. It was then stored at 4 °C and rapidly used for the oral infection. This strain was resistant to amoxicillin/clavulanic acid, streptomycin, nalidixic acid, gentamicin, doxycycline and sulfamethoxazole/trimethoprim and it was proven to harbor *invA*, *fliC*, *hilA*, *stn*, *pefA* and *sopB* virulence genes being a multidrug-resistant and multivirulent strain.

#### Birds inoculation

At d 23 of age, all experimental birds in PC, thymol and thymol nanoemulsion groups were challenged with the *S*. Typhimurium inoculum dose (one mL of 3 × 10^6^ CFU/bird), while NC group was not challenged. Individual birds were orally gavaged with *Salmonella* inoculum by a syringe with an affixed flexible tube, while the uninfected chickens were administered sterile Tryptone Soy Broth. The infection was checked till the end of the experiment (d 42) through re-isolation and identification of the challenging *Salmonella* strain from cecum of dead and euthanized birds in addition to re-examination of its antimicrobial susceptibility patterns and virulence genes.

#### Growth performance

Feed intake per each group and individual body weight (BW) were recorded to calculate the BWG (g/bird per day) and FCR at the end of starter, grower, and fisher periods. At the end of experiment, feed intake, BWG, and FCR were calculated for all rearing period (d 1–42) as previously described^79,80^.

### Sampling

#### Preinfection

At d 22 of age, cecal contents (n = 5/replicate) were collected and stored at − 80 °C till further bacteriological analysis. Cecal, pancreatic and splenic samples (n = 5/replicate) were taken as well into RNALater (Sigma, USA) for analysing the differential gene expressions of TJP, digestive enzymes, barrier functions and immune related parameters by the RT-qPCR assay.

#### Postinfection

Cecal contents (n = 5/replicate) were collected at d 30 (7 dpi) and 42 (14 dpi) of age and stored at − 80 °C for bacteriological examination, while cecal samples were used for quantitation of DNA copies of *S.* Typhimurium and analysis of mRNA expression of *S.* Typhimurium virulence gene (*invA*). Cecal, pancreatic and splenic samples (n = 5/replicate) were collected at d 42 for gene expression analysis of TJP, digestive enzymes, barrier functions and immune related parameters, respectively. Cecal and liver samples were collected for histopathological examination.

### Microbiological analyses

At 22, 30 and 42 d of age, birds were slaughtered (n = 5/replicate) and cecal contents were aseptically removed, weighed, and homogenized. Each homogenate was tenfold serially diluted in sterile phosphate-buffered saline. Appropriate dilutions were plated in duplicate for bacterial population counts using the surface drop technique^81^. Total aerobic bacterial counts were determined on Standard Methods Agar (Oxoid, UK) plates following aerobic incubation at 37 °C for 2–3 days. The number of anaerobic bacteria was detected on Plate Count Agar (Oxoid, UK) plates after anaerobic incubation at 35 °C for 48 h. Cecal bacteria in the family *Enterobacteriaceae* were enumerated on violet red bile dextrose agar (Oxoid, UK) plates incubated aerobically at 37 °C for 24 h. Total lactobacillus count was obtained on Rogosa agar (Oxoid, UK) plates after anaerobic incubation at 37 °C for 3 days. The average results of the duplicate measurements are presented as log_10_ colony forming units (CFU)/g of the cecal contents.

### Quantification of S. Typhimurium DNA copies

DNA from the cecal samples was extracted using a QIAamp DNA Stool Mini Kit (Qiagen GmbH, Germany) according to the manufacturer’s instructions. Extracted DNA concentrations and quality were assessed spectrophotometrically with a Spectrostar NanoDrop 2000 spectrophotometer (Thermo Fisher Scientific Inc., Waltham, MA, USA). Purified DNA was stored at − 80 °C for the subsequent quantitative PCR analysis. *Salmonella* numbers in cecal samples were determined by a real time PCR (RT-PCR) assay, which was carried out in 96-well polypropylene plates using a Stratagene MX3005P real time PCR machine. The PCR amplification was performed, in triplicate, in a total reaction volume of 25 µL consisting of 3 µL of DNA template, 12.5 µL of 2 × QuantiTect Probe RT-PCR Master Mix (Qiagen GmbH, Germany), 8.875 µL PCR grade water, 0.25 µL of 50 pmol concentration of each primer and 0.125 µL of 30 pmol concentration of the FAM-TAMRA labeled probe. The PCR primer and TaqMan probe sets targeting *invA* gene of *Salmonella* species and the respective PCR conditions were previously described in the quoted reference^82^. For quantification of target DNA copy numbers, a standard curve was constructed. The extracted DNA template from pure *S.* Typhimurium strain was tenfold serially diluted (6 times) and then quantified in a real time quantitative PCR run to determine the threshold cycle (Ct) value related to each dilution. The *Salmonella* concentration in each DNA sample was calculated by interpolating the Ct values of DNA samples from the cecal samples into the generated standard calibration curves and then their log_10_ of the CFU numbers were estimated.

### Gene expression analysis

Total RNA was extracted from chicken cecal, pancreatic and splenic samples according to the purification of total RNA from animal tissues protocol of QIAamp RNeasy Mini kit (Qiagen GmbH, Hilden, Germany). The concentration of the extracted RNA was assayed using a Spectrostar NanoDrop 2000 spectrophotometer (Thermo Fisher Scientific Inc., Waltham, MA, USA) at an optical density of 260 nm. The RNA purity was then verified by the ratio of absorbance at 260 nm and 280 nm. The expression levels of genes encoding TJP [occludin, zona occludens-1 (ZO-1), claudins-1 (CLDN 1) and junctional adhesion molecule (JAM)], digestive enzymes (AMY2A, PNLIP and CCK), barrier functions [fatty acid binding protein 2 (FABP2) and mucin-2 (MUC-2)], immune related parameters (IL-2, IL-6, IL-10 and IgA) and *S*. Typhimurium invasion protein A were determined by one step RT-qPCR assay using QuantiTect SYBR Green RT-PCR Kit (Qiagen GmbH, Hilden, Germany) on a Stratagene MX3005P real time PCR machine (Agilent Technologies, Inc., Santa Clara, CA, USA). All PCR reactions were applied in triplicate. After amplification, a final melting curve analysis was performed to investigate the presence or absence of the non-specific amplification products. The appropriate gene specific primer sets used in assessing the expression are characterized in Table 3. The expression levels of TJP, digestive enzymes and cytokines related genes were analyzed using glyceraldehyde-3- phosphate dehydrogenase (GAPDH) and TATA-binding protein (TBP) reference genes, while that of *invA* gene was normalized using *rpoD* as an internal housekeeping gene with the following sequence F: 5-ACATGGGTATTCAGGTAATGGAAGA-3 and R: 5-CGG TGCTGGTGGTATTTT CA-3 as described by Botteldoorn et al.^83^. The relative gene expression data were analyzed using the 2^−ΔΔCt^ method^84^.

**Table 3:** Primer sequences used for gene expression analysis by RT-qPCR assay.

Gene	Primer sequence (5′–3′)	Accession no
**Digestive enzymes**		
*AMY2A*	F-CGGAGTGGATGTTAACGACTGG R-ATGTTCGCAGACCCAGTCATTG	NM_001001473.2
*PNLIP*	F-GCATCTGGGAAG^↓^GAACTAGGG R- TGAACCACAAGCATAGCCCA	NM_001277382.1
*CCK*	F-AGGTTCCACTGGGAGGTTCT R-CGCCTGCTGTTCTTTAGGAG	XM_015281332.1
**Tight junction protein and gut barrier function**		
Occludin	F-ACGGCAAAGCCAACATCTAC R- ATCCGCCACGTTCTTCAC	XM_031604121.1
*ZO-1*	F-GCCAACTGATGCTGAACCAA R- GGGAGAGACAGGACAGGACT	XM_015278975
*CLDN 1*	F-GGTGAAGAAGATGCGGATGG R- TCTGGTGTTAACGGGTGTGA	NM_001013611
*MUC-2*	F-AAACAACGGCCATGTTTCAT R- GTGTGACACTGGTGTGCTGA	NM_001318434
*JAM-2*	F-AGACAGGAACAGGCAGTGCT R- TCCAATCCCATTTGAGGCTA	XM_031556661.1
*FABP2*	F-AGGCTCTTGGAACCTGGAAG R- CTTGGCTTCAACTCCTTCGT	NM_001007923
**Immune related genes**		
*IL-2*	F: GCTTATGGAGCATCTCTATCATCA R: GGTGCACTCCTGGGTCTC	XM_015276098.2
*IL-6*	F: AGGACGAGATGTGCAAGAAGTTC R: TTGGGCAGGTTGAGGTTGTT	NM_204628.1
*IL-10*	F: GCTGAGGGTGAAGTTTGAGG R: AGACTGGCAGCCAAAGGTC	XM_025143715.1
*IgA*	F: ACCACGGCTCTGACTGTACC R: CGATGGTCTCCTTCACATCA	S40610.1
**House keeping**		
*GAPDH*	F: CAACCCCCAATGTCTCTGTT R: TCAGCAGCAGCCTTCACTAC	NM205518
*TBP*	F: GTCCACGGTGAATCTTGGTT R: GCGCAGTAGTACGTGGTTCTC	Acc:8484
***Salmonella Typhimurium***		
*invA*	F: GTGAAATTATCGCCACGTTCGGGCAA R: TCATCGCACCGTCAAAGGAACC	KF026356

*AMY2A*: pancreatic alpha 2A amylase; *PNLIP*: pancreatic lipase; *CCK*: cholecystokinin; *ZO-1*: zonula occludens; *CLDN 1:* claudins-1; *MUC-2:* Mucin-2; *JAM-2*: junctional adhesion molecules-2; *FABP2*: fatty acid binding proteins2; *IL*-2: interleukin-2; *IL -6*: interleukin-6; *IL-10*: interleukin-10; *IgA*: immunoglobulin A; *GAPDH*: glyceraldehyde-3-phosphate dehydrogenase; *TBP*: TATA-binding protein; *invA*: *S*. Typhimurium invasion protein A gene.

### Histological/histopathological examination

At the end of the study (at 14 dpi), samples from the liver and cecum of slaughtered birds (n = 5/group) were obtained for the evaluation of the histopathological alterations in the studied groups. Liver samples were immediately collected post-slaughter, while the histological preparation of the intestinal specimens was performed as we previously reported^85^. Briefly, a piece from the middle portion of the cecum was obtained and flushed with warm physiological saline and freshly prepared 10% neutral buffered formalin solution. Then, liver and cecum samples were immediately fixed in freshly prepared 10% neutral buffered formalin for at least 48 h. Afterwards, standard histological technique was applied (that is dehydration in ascending grades of alcohol, clearing in xylene and paraffin wax embedding). Thin sections (5 μm) were cut and stained with H&E stain and examined under light microscopy. Stained slides were blindly evaluated by two independent pathology experts and the lesions were detected and documented.

### Statistical analysis

The analysis for all data was performed using the GLM model procedure after confirming the homogeneity among experimental groups using Levene’s test and normality using Shapiro–Wilk’s test. Significance (*p* < 0.05) among treatments was determined by the Tukey test.

### Ethical approval

The care and use of chickens in the present study were performed in compliance with the guidelines and regulations approved by the Institutional Animal Care and Use Committee (ZU-IACUC/6/f/2019), Poultry Research Unit, Faculty of Veterinary Medicine, Zagazig University. All animal protocols were carried out in compliance with the ARRIVE guidelines.

## Supplementary Information

Supplementary Information

## Data Availability

The datasets generated and/or analysed during the current study are available from the corresponding author on reasonable request.
