# Supplementary material for: Thymol nanoemulsion promoted broiler chicken’s growth, gastrointestinal barrier and bacterial community and conferred protection against Salmonella Typhimurium
Source: Sci Rep. 2021 Apr 8;11:7742. doi: 10.1038/s41598-021-86990-w (PMC8032708; doi:10.1038/s41598-021-86990-w)
Supplement: Supplementary file 1 — Supplementary Information [file 41598_2021_86990_MOESM1_ESM.docx]

**Supplementary data**

**Thymol nanoemulsion promoted broiler chicken’s growth, gastrointestinal barrier and bacterial community and conferred protection against *Salmonella* Typhimurium**

**Doaa Ibrahim ^1*^, Ahmed Abdelfattah-Hassan^2,3^, M. Badawi ^1^, Tamer Ahmed Ismail ^4^, Mahmoud. M. Bendary ^5^, Adel M. Abdelaziz ^6^ , Rasha A. Mosbah ^7^, Dalia Ibrahim Mohamed ^8^ , Ahmed. H. Arisha^9, 10^, Marwa I. Abd El-Hamid ^11*^**

^1^ Department of Nutrition and Clinical Nutrition, Faculty of Veterinary Medicine, Zagazig University, Egypt.

^2^ Department of Anatomy and Embryology, Faculty of Veterinary Medicine, Zagazig University, Egypt

^3^ Biomedical Sciences Program, University of Science and Technology, Zewail City of Science and Technology, October Gardens, 6^th^ of October, Giza 12578, Egypt

^4^ Department of Clinical Laboratory Sciences, Turabah University College, Taif University, P.O. Box 11099, Taif 21944, Saudi Arabia.

^5^ Department of Microbiology and Immunology, Faculty of Pharmacy, Port Said University, Port Said Governorate, Egypt

^6^ Veterinary Educational Hospital, Faculty of Veterinary Medicine, Zagazig University, Egypt.

^7^ Fellow Pharmacist at Zagazig University Hospital, Zagazig, Egypt

^8^ Department of Biochemistry, Animal Health Research Institute, Zagazig Branch, Agriculture Research Center, Egypt.

^9^ Department of physiology, Faculty of Veterinary Medicine, Zagazig University, Egypt.

^10^ Department of Animal Physiology and Biochemistry, Faculty of Veterinary Medicine, Badr University in Cairo (BUC), Badr City, Cairo, Egypt.

^11^ Department of Microbiology, Faculty of Veterinary Medicine, Zagazig University, Zagazig, Egypt

Corresponding author: Doaa Ibrahim ([doibrahim@vet.zu.edu.eg](mailto:doibrahim@vet.zu.edu.eg)).

Marwa I. Abd El-Hamid ([mero_micro2006@yahoo.com](mailto:mero_micro2006@yahoo.com)).

**Phagocytic index in response to thymol and thymol nanoemuslion** **feeding**

For phagocytosis assay, heparinized blood samples were randomly collected from birds` wing veins taken at the 14^th^ day post thymol and thymol nanoemuslion feeding for separation of peripheral blood mononuclear cells (PMNCs) using a Ficoll-Paque separation media (Gibco, United Kingdom) with a density of 1.077 g/mL. The chicken PMNCs were washed twice with phosphate-buffered saline (PBS) and suspended in RPMI 1640 medium (Gibco, USA) containing heat-inactivated autologous serum and antibiotics (100 µg streptomycin and 100 IU penicillin / mL) (Oxoid, UK). The cell viability was assessed using the trypan-blue exclusion test (viabilily always more than 95%) and then the viable cells allowed to adhere onto a sterile glass coverslip in a Petri dish was adjusted to be 2x10^5^ leukocytes/mL. After two hours incubation in the presence of 5% CO_2_ at 40°C, the coverslips were rinsed with PBS to remove the nonadherent cells. The adherent cells were incubated with a *S*. Typhimurium suspension of 1x10^6^/well in RPMI 1640 at 37°C in a humidified CO_2_ incubator for 40 minutes. The coverslips were then washed with RPMI 1640 to get rid of the non-phagocytosed bacteria, fixed with absolute methanol and stained with 20% buffered Giemsa solution. The number of ingested and/or attached *S*. typhimurium per 200 macrophages, in duplicates, preparations, was microscopically assessed. The phagocytic index was calculated as the average number of bacteria engulfed by macrophages multiplied by the percentage of these cells engaged in phagocytosis^1^.

**Result and discussion**

Macrophages from bird groups supplemented with thymol and thymol nanoemuslion exhibited improvement in the phagocytosis and phagocytic index value at the 14^th^ day post feeding compared to the negative control group (Supplementary Figure 1). The increase in the phagocytic index (up to 645) was exclusively due to the enhancement in the average number of phagocytosed bacteria by individual macrophage (up to 6.8) and the increase in the percentage of macrophages engaged in phagocytosis (up to 95). Chicks receiving 1% thymol and 0.5% and 1% thymol nanoemulsion had higher significant phagocytic index (492 to 645; p<0.05) compared to the negative control group. Supplementation of a base diet with thyme oil improved the phagocytic activity in blood, which translates into a better immune response of broiler chickens^2^. In another study conducted in Korea, thymol augmented phagocytosis by enhancing the macrophages membrane fluidity due to its potent immunostimulating effect^3^. The observed increase in phagocytosis appears to be due to the increased expression of the receptors on the macrophages plasma membranes for the Fc portion of immunoglobulins mediating and enhancing the phagocytosis and associated killing mechanisms.

References:

1 Muniz-Junqueira, M. I., Prata, A. & Tosta, C. E. Factors influencing phagocytosis of *Salmonella* typhimurium by macrophages in murine schistosomiasis. *Rev. Soc. Bras. Med. Trop.* **30**, 101-106 (1997).

2 Placha, I. *et al.* Effect of thyme essential oil and selenium on intestine integrity and antioxidant status of broilers. *Br. Poult. Sci.* **55**, 105-114 (2014).

3 Chauhan, A. K., Jakhar, R., Paul, S. & Kang, S. C. Potentiation of macrophage activity by thymol through augmenting phagocytosis. *Int. Immunopharmacol.* **18**, 340-346 (2014).

**Figure 1.** Macrophage % (a), average of bacteria engulfed by macrophages (b) and phagocytic index (c) of broiler chickens fed different levels of thymol and thymol nanoemulsion postinfection.

Values are means with their SE in bars. Bars with different letters denote significant differences among different groups (p<0.05).
